# A comparative assessment of adipose‐derived stem cells from subcutaneous and visceral fat as a potential cell source for knee osteoarthritis treatment

**DOI:** 10.1111/jcmm.13138

**Published:** 2017-04-04

**Authors:** Yan Tang, Zhang‐yi Pan, Ying Zou, Yi He, Peng‐yuan Yang, Qi‐qun Tang, Feng Yin

**Affiliations:** ^1^ Institute of Stem Cell Research and Regenerative Medicine Institutes of Biomedical Sciences Fudan University Shanghai China; ^2^ Department of Joint Surgery Shanghai East Hospital School of Medicine Tongji University Shanghai China; ^3^ Department of Cardiothoracic Surgery Xinhua Hospital Shanghai Jiaotong University of Medicine College Shanghai China; ^4^ Translational Medical Center for Stem Cell Therapy Shanghai East Hospital Tongji University Shanghai China

**Keywords:** subcutaneous adipose‐derived stem cells, visceral adipose‐derived stem cells, osteoarthritis

## Abstract

The intra‐articular injection of adipose‐derived stem cells (ASCs) is a novel potential therapy for patients with osteoarthritis (OA). However, the efficacy of ASCs from different regions of the body remains unknown. This study investigated whether ASCs from subcutaneous or visceral adipose tissue provide the same improvement of OA. Mouse and human subcutaneous and visceral adipose tissue were excised for ASC isolation. Morphology, proliferation, surface markers and adipocyte differentiation of subcutaneous ASCs (S‐ASCs) and visceral ASCs (V‐ASCs) were analysed. A surgically induced rat model of OA was established, and 4 weeks after the operation, S‐ASCs, V‐ASCs or phosphate‐buffered saline (PBS, control) were injected into the articular cavity. Histology, immunohistochemistry and gene expression analyses were performed 6 weeks after ASC injection. The ability of ASCs to differentiate into chondrocytes was assessed by *in vitro* chondrogenesis, and the immunosuppressive activity of ASCs was evaluated by co‐culturing with macrophages. The proliferation of V‐ASCs was significantly greater than that of S‐ASCs, but S‐ASCs had the greater adipogenic capacity than V‐ASCs. In addition, the infracted cartilage treated with S‐ASCs showed significantly greater improvement than cartilage treated with PBS or V‐ASCs. Moreover, S‐ASCs showed better chondrogenic potential and immunosuppression *in vitro*. Subcutaneous adipose tissue is an effective cell source for cell therapy of OA as it promotes stem cell differentiation into chondrocytes and inhibits immunological reactions.

## Introduction

OA is a common form of chronic degenerative joint disease that is slowly induced in the bone, synovium and muscle by several processes including progressive cartilage deterioration, subchondral bone remodelling, loss of joint space, marginal osteophytosis and loss of joint function 1, 2. Current treatment options for articular injury, for instance, physical therapy and anti‐inflammatory drugs, aim to remedy the symptoms, but they do little to treat the underlying causes 3. When symptomatic medical treatments fail, patients usually resort to receiving autologous chondrocyte implantation (ACI) and total knee arthroplasty (TKA) 4, 5. ACI can repair cartilage, but often leads to insufficient results due to the poor self‐renewal potential of chondrocytes 5. TKA is a surgical procedure in which the dysfunctional joint surface is replaced with an orthopaedic prosthesis 4. Both ACI and TKA surgeries carry relatively high morbidity and mortality rates. In recent years, researchers have focused on less invasive treatments to repair full‐thickness articular cartilage, such as the use of mesenchymal stem cells (MSCs).

MSCs are multipotent progenitor cells that were originally identified within the bone marrow in 1967 6, 7. They have been applied for the treatment of OA in clinical trials because of their regeneration potential and anti‐inflammatory effects 8. The first reported use of bone marrow stem cells (BMSCs) to repair cartilage damage in humans was in 1998 9. Subsequently, researchers have found that the use of BMSCs is an effective and safe way of treating cartilage defects in most cases 10. However, the low stem cell yield requiring long expansion time *in vitro*, pain and possible morbidities during bone marrow aspiration restrict their use in clinical therapy. ASCs are thought to be more suitable in clinical application because of the high stem cell yield from lipoaspirates, faster cell proliferation and less discomfort and morbidities during the harvesting procedure. In addition, these adult stem cells are capable of differentiating into multiple cell types and thus are useful in regenerative medicine. In 2001 and 2002, Zuk *et al*. 11, 12 isolated ASCs from human adipose tissue and showed that they have multipotential capacity. The clinical use of ASCs was demonstrated in a case study reported by Pak in 2011 13, in which intra‐articular injection of autologous ASCs (approximately 100 g adipose tissue) from the abdomen with platelet‐rich plasma, hyaluronic acid and dexamethasone was used to treat OA. In 2012, Koh and Choi 14 used ASCs obtained from the knee fat pad (approximately 20 g) and found that less than 2 million ASCs could cause cartilage regeneration. Therefore, adipose tissue from different regions of the body may contain different numbers of stem cells.

In humans and animals, there are two main types of white adipose tissue (WAT): subcutaneous (SC) and visceral (VS) adipose tissue, which differ in their pathophysiological properties including insulin sensitivity, adipokine secretion, lipolysis and development of inflammation 15. ASCs from SC and VS depots have intrinsic differences *in vitro* such as proliferation and differentiation potentials 16. ASCs derived from SC fat easily differentiate into mature adipocytes, whereas VS derived from ASCs differentiate poorly in response to a standard induction cocktail 17. Many studies have suggested that S‐ASCs could be a stem cell source for treating knee OA 18, 19. However, recently, VS adipose tissue has drawn a great deal of attention with regard to its differences from SC adipose tissue. In 2009, Baglioni and colleagues 20 successfully isolated a population of adult stem cells from the omental adipose tissue of human patients. Subsequently, several studies have shown that S‐ASCs and visceral ASCs (V‐ASCs) have differences in gene expression, adiponectin release and insulin signalling 20, 21, 22. However, researchers found that both SC and VS adipose tissues are equally effective cell sources for the treatment of heart failure 23. These observations led us to investigate whether S‐ASCs and V‐ASCs are equally effective in improving OA.

Mouse and human SC and VS adipose tissue were excised for isolation of ASCs. Morphology, proliferation, surface markers and adipocyte differentiation of S‐ASCs and V‐ASCs were analysed. A surgically induced rat model of OA was established, and 4 weeks after the operation, S‐ASCs, V‐ASCs and PBS, control were injected into the articular cavity. Histology, immunohistochemistry (IHC) and gene expression analyses were performed 6 weeks after ASC injection. In addition, the ability of ASCs to differentiate into chondrocytes was assessed and the immunosuppressive activity of ASCs was evaluated by co‐culturing with macrophages. The proliferation of V‐ASCs was significantly greater than that of S‐ASCs, but S‐ASCs had the greater adipogenic capacity than V‐ASCs. In addition, infarcted cartilage treated with S‐ASCs had significantly greater improvement than cartilage treated with PBS or V‐ASCs. Moreover, S‐ASCs showed better chondrogenic potential and immunosuppression *in vitro*. In conclusion, SC adipose tissue is an effective cell source for cell therapy of OA as it promotes stem cell differentiation into chondrocytes and inhibits immunological reactions.

## Materials and methods

### Animals

Animal handling and experimental procedures were performed following approval from the Institute of Health Sciences Institutional Animal Care and Use Committee. The 8‐week‐old Sprague Dawley rats (200 g) were randomized into four groups (*n* = 10 rats per group): sham group, OA group, S‐ASC treatment group and V‐ASC treatment group. OA was induced by medial collateral ligament (MCL) transection and medial meniscal tear of the right knee joints. Briefly, the animals were anesthetized, and surgery was performed to transect the MCL. The medial meniscus was cut across the full thickness to induce joint destabilization of the right knee (OA group). Sham animals underwent the same surgical procedure, but without ligament transection or meniscal tear. After surgery, each rat was given penicillin once a day for the first 3 days. For stem cell treatment, S‐ASCs or V‐ASCs (4 × 10^6^ cells/rat) were directly injected into the intra‐articular space of the knee joints of recipient rats 4 weeks after surgery. The rats were euthanized 10 weeks after surgery, and knee joint samples were collected and fixed in 4% paraformaldehyde overnight, followed by a decalcifying step in 4% EDTA for at least 1 month. Fluid was changed every 2 days and embedded in paraffin. Sections (4 μm thick) of the medial compartment of the knee joint were processed for haematoxylin and eosin staining, safranin O staining and IHC.

### Isolation and culture of ASCs

S‐ASCs and V‐ASCs were obtained from 3‐month‐old and 10‐month‐old male mice according to standard procedures. S‐ASCs and V‐ASCs were also obtained from healthy volunteers. The age range of the subjects was 23–49 years. Briefly, stem cells were prepared from WAT by incubating in a solution containing 0.075% collagenase (Sigma‐Aldrich, St. Louis, Missouri, USA) for 40 min. followed by centrifugation at 200 g for 5 min. The freshly isolated cells were plated in F12 medium supplemented with glutamax, penicillin/streptomycin and 10% foetal bovine serum (FBS, Gibco, Grand Island, NY, USA.) and maintained in a 5% CO_2_ atmosphere. The study protocol was reviewed and approved by the Ethics Committee of Fudan University, and subjects provided written, informed consent following the committee's instructions. The study was conducted according to the principles of the Declaration of Helsinki.

### Flow cytometry

The stromal vascular fraction (SVF) from the WAT of 3‐month‐old mice was isolated as described above and resuspended in PBS with 0.5% FBS. The PE‐anti mouse CD34 antibody (BioLegend, San Diego, CA, USA) was used and antibody incubations were performed on ice for 45 min., after which the cells were washed and resuspended in PBS with 2% paraformaldehyde for analysis. The samples were analysed on a BD Accuri C6 flow cytometer, and 5‐ethynyl‐2′‐deoxyuridine (EdU) incorporation was analysed using the Alexa Fluor‐647EdU Flow Kit (Life Technologies, Carlsbad, California, USA.) according to the manufacturer's protocol.

### Adipocyte differentiation assays

The freshly isolated cells were plated in F12 medium supplemented with glutamax, penicillin/streptomycin and 10% FBS (Gibco) and maintained in a 5% CO2 atmosphere. The cells were allowed to grow to confluence and then were maintained at confluence for 2 days without changing the medium prior to treatment with a differentiation cocktail (5 g/ml insulin, 1 M dexamethasone, 0.5 mM IBMX [a phosphodiesterase inhibitor], 1 M rosiglitazone for 48 hrs, then 5 g/ml insulin and 1 M rosiglitazone for 48 h). After exposure to the differentiation cocktail for 4 days, the cells were maintained in F12 medium with 10% FBS until harvesting on day 8. For staining, the cells were fixed in 4% formaldehyde in PBS for 15 min., rinsed with water, stained with Oil Red O for 1 hr and then rinsed with water again.

### Aggregate chondrogenesis model

ASCs were centrifuged into cell aggregates and induced along the chondrogenic lineage. Cells were suspended to a concentration of 1 × 10^7^ cells/ml, seeding 5 μl droplets of cell solution in the centre of the six‐well plates. After cultivating the micromass cultures for 2 hrs under high humidity conditions, warmed chondrogenesis media were added to the culture vessels and incubated at 37°C with 5% CO_2_. Then, the supernatant was aspirated and replaced with chondrogenic inductive medium (CIM) consisting of F12/DMEM (containing L‐glutamine and sodium pyruvate), 1% Ab/Am cocktail, 1% ITS+Premix (Life Technologies), 40 μg/ml proline, 100 nM dexamethasone, 50 μg/ml ascorbic acid‐2‐phosphate (Sigma‐Aldrich) and 10 ng/ml rhTGF‐β1 (Peprotech). Media were changed every other day, and aggregates were collected at various time‐points for analysis, as described below.

### IHC

IHC was performed according to the Vectastain Elite ABC kit protocols (Vector Laboratories, Burlingame, CA, USA), and antibody staining was visualized with the enhanced DAB kit (Vector Laboratories). The brown reaction product was quantified using Image‐Pro Plus software.

### RNA extraction

Complementary DNA synthesized from total RNA was analysed in a Sequence Detector (Q5; Bio‐Rad, Hercules, California, USA) with specific primers and SYBR Green PCR Master reagents (ABI). The relative abundance of mRNA was calculated with 18S mRNA as the invariant control. The primers were derived from Primer Bank (http://pga.mgh.harvard.edu/primerbank/), and the primer sequences are reported in Table S1.

### Antibodies and immunoblotting

The total protein extractions were homogenized in lysis buffer and loaded onto a gel for electrophoresis. Then, the proteins were transferred onto nitrocellulose membranes that were subsequently immunoblotted with specific antibodies against Sox9 (Santa Cruz Biotechnology, Delaware Ave, CA, USA.) and actin (Sigma‐Aldrich).

### Statistical analysis

All of the results were presented as mean ± standard error of the mean (S.E.M.). Student's *t*‐test was used for the statistical analyses, and *P* values less than 0.05 were considered statistically significant.

## Results

### Characteristics of ASCs from SC and VS adipose tissue

**Figure 1:**
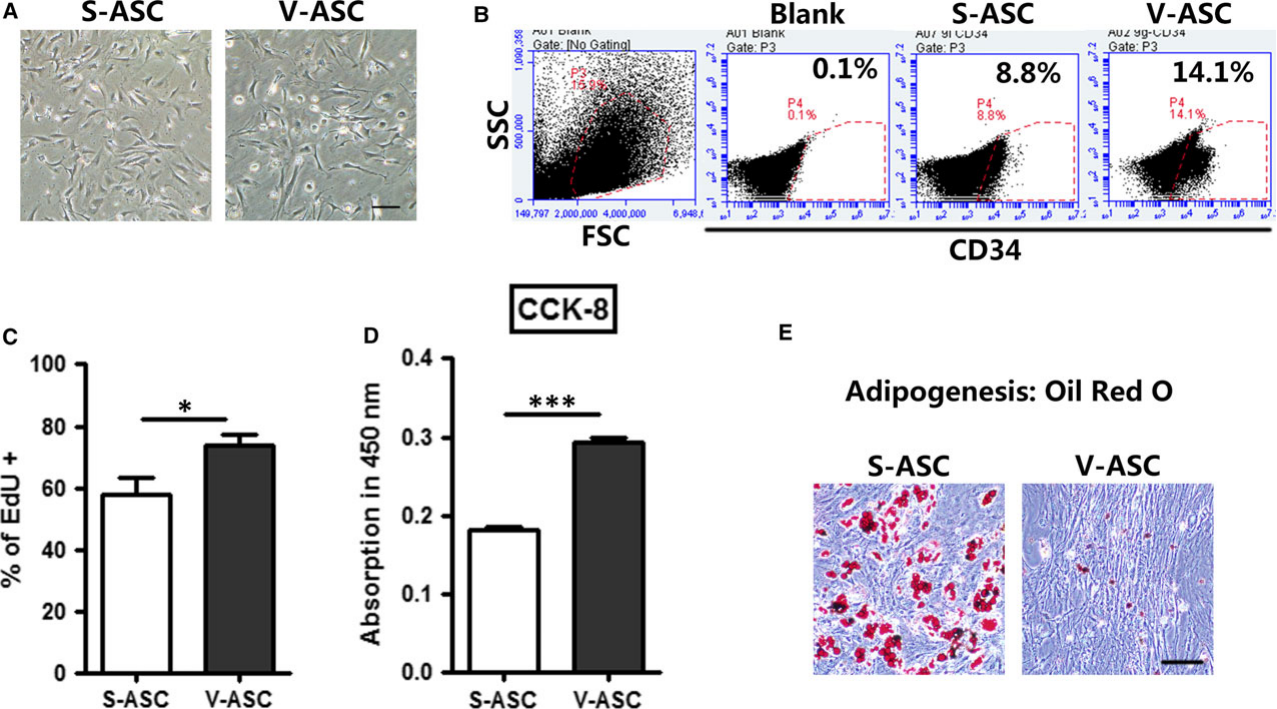
Characterization of stem cells from SC adipose tissue and VS adipose tissue. (**A**) Morphology of cultured S‐ASCs and V‐ASCs in passage P2 from 10‐month‐old WT C57 mice. Scale bar = 200 μm; (**B**) flow cytometric analysis of CD34^+^ stem cells in the SVF of the inguinal adipose tissue (SC) and gonadal adipose tissue (VS) from 10‐month‐old WT C57 mice; (**C**) primary ASCs were incubated with EdU (10 μM) for 24 hrs and EdU+ cells were analysed by flow cytometry; (**D**)the CCK‐8 assay was performed to compare cell proliferation; (**E**) Oil Red O staining of adipocytes differentiated from S‐ASCs and V‐ASCs on day 8. Scale bar = 100 μm. *P < 0.05, **P < 0.01.

Following initial isolation and expansion, homogeneous ASCs growing in a monolayer with a spindle‐shaped morphology were observed after culture for 2 days. ASCs isolated from both SC and VS adipose tissue exhibited typical fibroblast‐like spindle morphology (Fig. 1A). In addition, both cell types displayed positive staining for the mesenchymal surface marker CD34; however, the expression of CD34 in V‐ASCs was higher than that in S‐ASCs (Fig. 1B). This suggests that the two types of ASCs share common morphological, but different biological properties. The two types of ASCs presented with strong proliferation capacity *in vitro*. The ASCs reached 80–90% confluence 2 days after initial seeding for the first passage. To assess their proliferation rate, EdU incorporation and CCK‐8 assays were performed. The results revealed that the proliferation capacity of V‐ASCs was stronger than that of S‐ASCs (Fig. 1C and D). To further investigate the adipocyte differentiation capacity of ASCs derived from SC or VS adipose tissue, cells were cultured in adipogenic differentiation media and adipogenesis was observed by staining with Oil Red O. S‐ASCs showed higher adipogenic ability compared to V‐ASCs (Fig. 1E). These results indicate that S‐ASCs are less capable of proliferation, but are more able to differentiate into mature adipocytes.

### Intra‐articular injection of S‐ASCs inhibit OA progression

**Figure 2:**
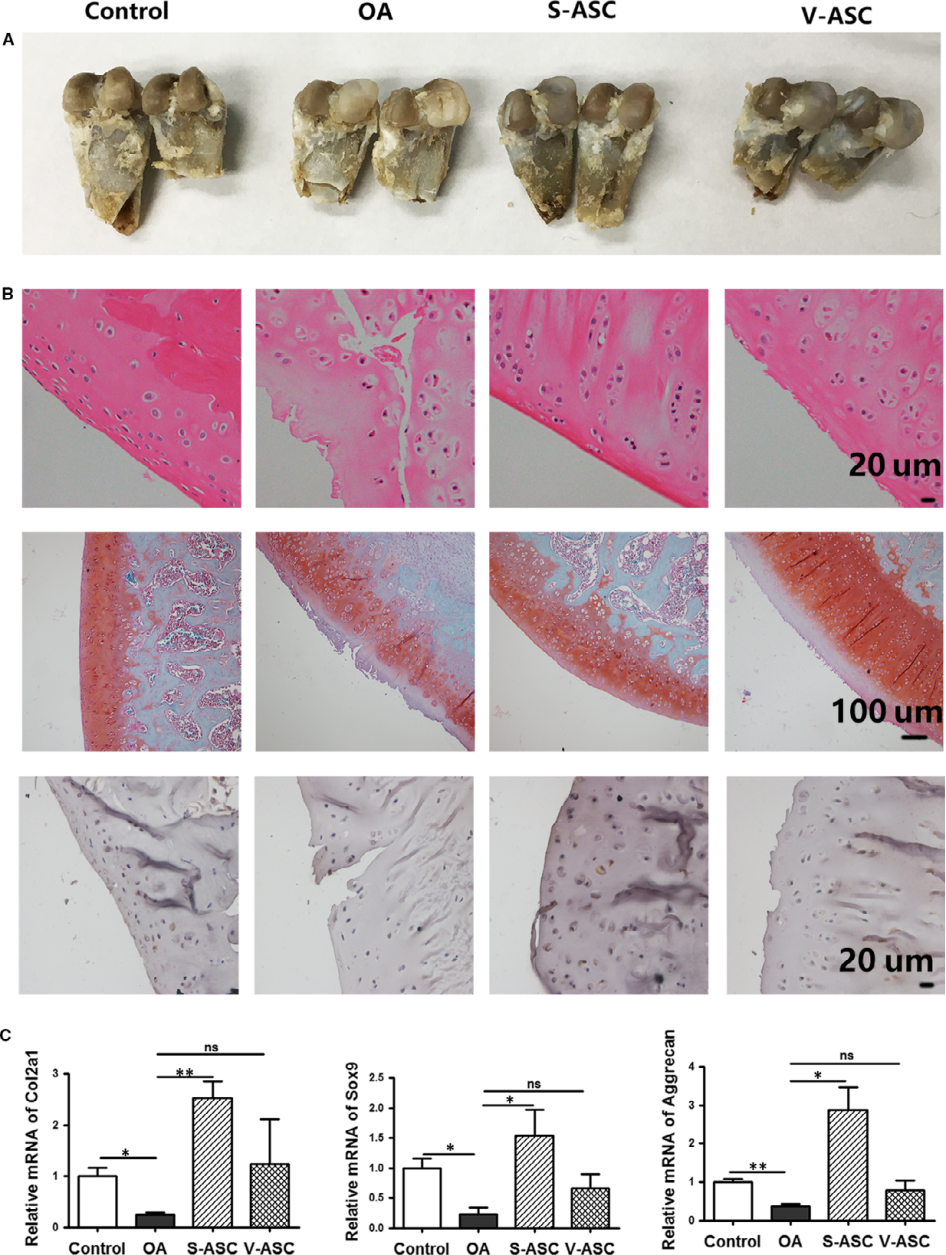
Effects of S‐ASCs and V‐ASCs in the rat model of OA. OA was created in the right knee of Sprague Dawley rats. ASC‐treated cartilage was compared to OA rat models treated with PBS. (**A**) Representative gross morphologies; (**B**) staining results of haematoxylin and eosin, safranin O and IHC for type‐II collagen; (**C**) articular cartilage from the right knee in the four treatment groups was used for qPCR analysis to determine the relative mRNA expression of the indicated chondrocyte‐related markers (*n* = 6). *P < 0.05, **P < 0.01.

Studies have shown that the intra‐articular injection of autologous ASCs from SC adipose tissue or infrapatellar fat into the osteoarthritic knee improved function and pain of the knee joint in humans 14, 18, 19, suggesting that ASCs from different regions of the body may all have cartilage repair functions. To evaluate the therapeutic efficacy of S‐ASCs and V‐ASCs, we administered intra‐articular injections of S‐ASCs and V‐ASCs into a surgically induced OA rat model to compare their effects. Gross morphology demonstrated alleviated osteophyte and fibrous tissue formation in the tibia cartilage upon S‐ASC treatment, compared to treatment with PBS or V‐ASCs (Fig. 2A). Histological analysis of control rats showed fibrotic tissue and damaged cartilage surface, whereas rats treated with S‐ASCs had a smooth cartilage surface as well as distribution of lacunae and chondrocytes. Additionally, immunostaining of Acan and Collagen type‐II alpha (Col2A1) showed enhanced expression in the cartilage upon S‐ASC treatment (Fig. 2B). The expression levels of several chondrogenesis‐related markers, including Col2A1, Sox9 and Aggrecan, were evaluated by real‐time PCR (qPCR), and the results showed that the mRNA expression of these genes was higher in the S‐ASC treatment group than in the OA and V‐ASC treatment groups (Fig. 2C). Taken together, these *in vivo* data demonstrate that compared to V‐ASCs, S‐ASC treatment delayed cartilage degradation in the rat model of OA.

### Chondrogenic gene expression and cartilage phenotype differentiation of S‐ASCs and V‐ASCs *in vitro*

**Figure 3:**
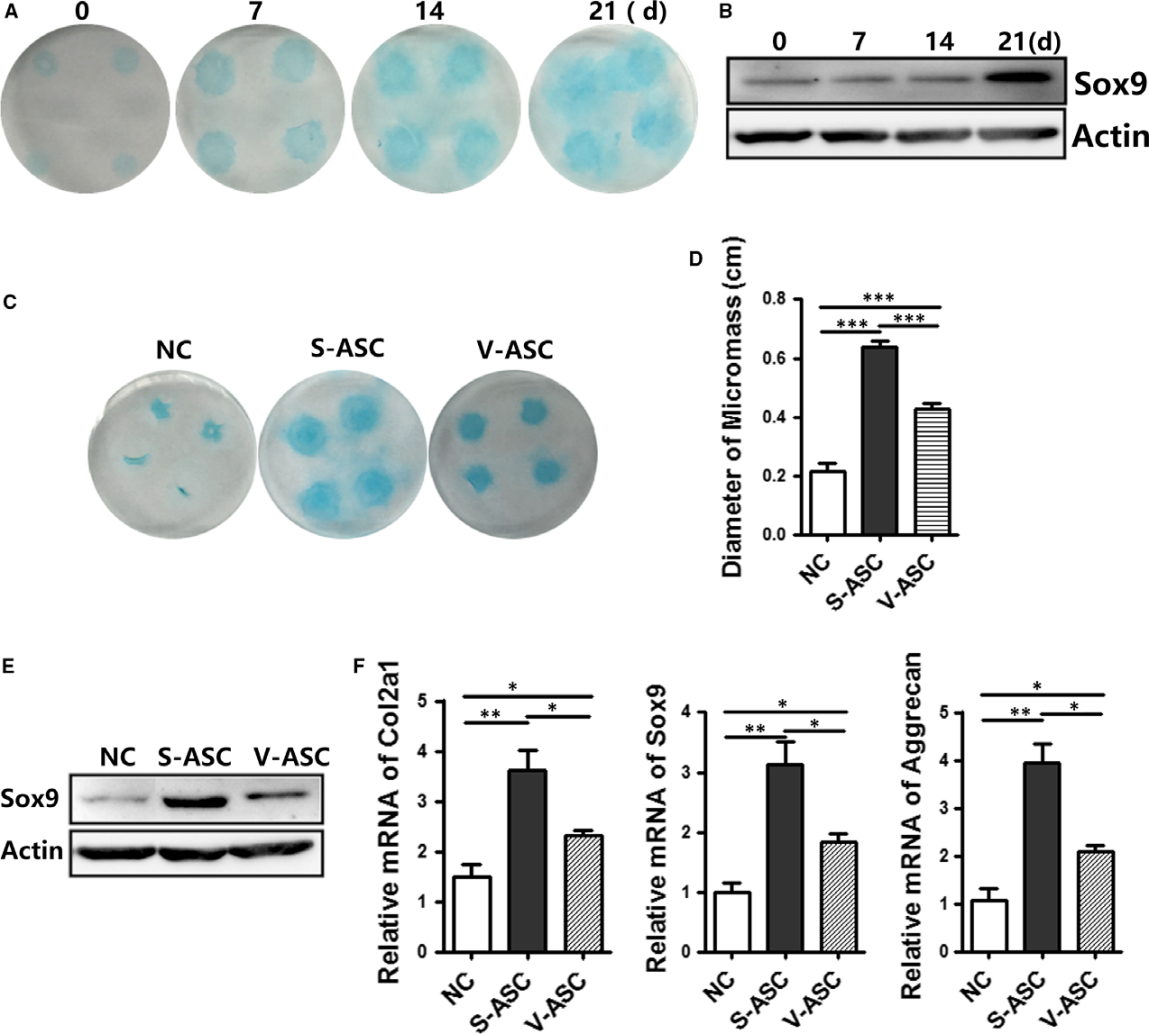
Chondrogenesis of stem cells from SC adipose tissue and VS adipose tissue in micromass cultures. (**A** and **B**) S‐ASCs in micromass cultures were treated with chondrogenic media at different times. Cultures were stained with Alcian blue (**A**) and Western blot analysis of the lysates (40 μg) (**B**) (**C–F**) the chondrogenesis capabilities of S‐ASCs and V‐ASCs were evaluated by Alcian blue staining (**C**); diameter of the micromass; (**D**) (*n* = 4), Western blot analysis of lysates (40 μg); (**E**) and relative mRNA expression of the indicated chondrocyte‐related markers (**F**) (*n* = 3) with non‐differentiated or differentiated medium on day 21. *P < 0.05, **P < 0.01, ***P < 0.001.

MSCs are able to directly promote tissue repair, in part because of their biodiverse functionalities 24, 25, 26. The micromass culture system is a classical model used to investigate chondrogenesis *in vitro*. We used Alcian blue to confirm the upregulation of Sox9 in micromass cultures (Fig. 3A and B). The chondrogenic differentiation capability of S‐ASCs was greater than that of V‐ASCs, as shown by Alcian blue staining and the micromass diameter (Fig. 3C and D). Western blot analyses showed that Sox9 protein expression was higher in S‐ASCs than in V‐ASCs (Fig. 3E). The gene expression profile of the three cartilage markers (Col2A1, SOX9 and aggrecan) was quantified by qPCR, and the results showed that their levels were higher in S‐ASCs than in V‐ASCs (Fig. 3F). This phenomenon suggests that S‐ASCs have pluripotent differential potential, but are unevenly distributed and prone to chondrogenesis.

### Chondrogenic gene expression and cartilage phenotype differentiation from human S‐ASCs and V‐ASCs *in vitro*

**Figure 4:**
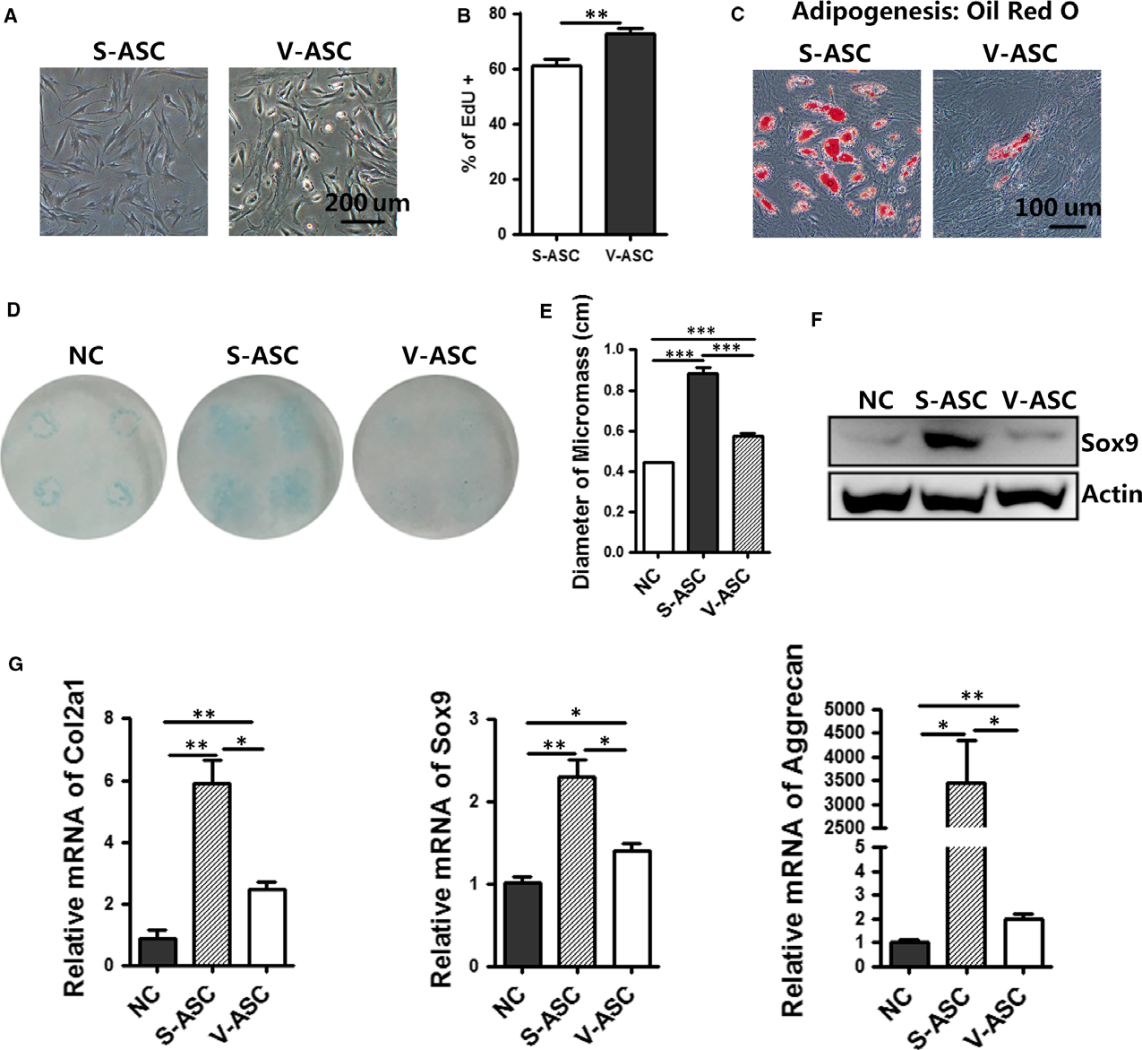
Chondrogenic gene expression and cartilage phenotype differentiation of human S‐ASCs and V‐ASCs *in vitro*.(**A**) Morphology of cultured human abdominal S‐ASCs and V‐ASCs in passage P2. Scale bar = 200 μm; (**B**) primary ASCs were incubated with EdU (10 μM) for 24 hrs and EdU+ cells were analysed by flow cytometry; (**C**) Oil Red O staining of adipocytes differentiated from S‐ASCs and V‐ASCs on day 8. Scale bar = 100 μm (**D–G**). The chondrogenesis capabilities of human S‐ASCs and V‐ASCs were evaluated by Alcian blue staining (**D**), diameter of the micromass (**e**) (*n* = 4), Western blot analysis of lysates (40 μg) (**F**) and relative mRNA expression of the indicated chondrocyte‐related markers (**G**)(*n* = 3) with non‐differentiated or differentiated medium on day 21. *P < 0.05, **P < 0.01, ***P < 0.001.

To determine whether human S‐ASCs are more effective than V‐ASCs in treating OA, we isolated primary S‐ASCs and V‐ASCs from the same patient undergoing gastrointestinal operation. These two types of ASCs displayed the same fibroblast‐like morphology (Fig. 4A). The results of the EdU incorporation assay showed that V‐ASCs had higher proliferation ability compared to S‐ASCs, which was consistent with the findings in mouse (Fig. 4B). After adipocyte cocktail treatment, Oil Red O staining of S‐ASCs showed higher adipogenic ability compared with that of V‐ASCs (Fig. 4C). To compare their ability to treat OA, *in vitro* chondrogenesis was assessed by histological staining with Alcian blue (Fig. 4D) and the micromass diameter was measured (Fig. 4E). The results showed that S‐ASCs had a twofold while V‐ASCs had a 1.2‐fold wider diameter than non‐chondrogenesis media. Additionally, qPCR and Western blot analyses showed increased expression of chondrocyte‐specific markers (col2a1, sox9, aggrecan) from S‐ASCs (Fig. 4F and G).

### Anti‐inflammatory effects of S‐ASCs in a lipopolysaccharide‐induced inflammatory model

**Figure 5:**
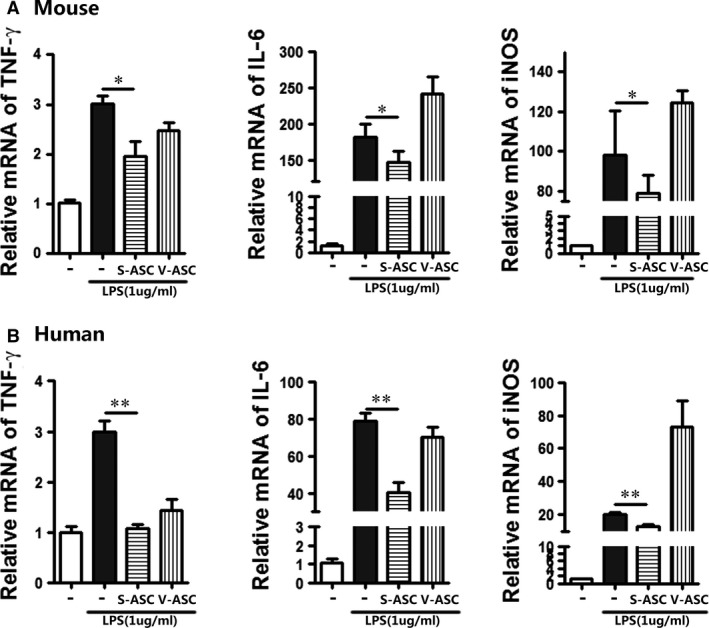
Effects of supernatant from S‐ASCs and V‐ASCs on TNF‐α, IL‐6 and iNOS expressions in LPS‐induced RAW 264.7 cells.RAW 264.7 macrophages were stimulated with LPS and cultured with supernatant from S‐ASCs and V‐ASCs for 24 hrs. (**A**) Relative mRNA expression of the indicated pro‐inflammatory cytokines in RAW 264.7 cells treated with F12/DMEM or supernatant from mouse S‐ASCs and V‐ASCs was determined by qPCR (*n* = 3); (**B**) relative mRNA expression of the indicated pro‐inflammatory cytokines in RAW 264.7 cells treated with F12/DMEM or supernatant from human S‐ASCs and V‐ASCs was determined by qPCR (*n* = 3). *P < 0.05, **P < 0.01.

In addition to the potential for ASCs to differentiate into chondrocytes, their ability to promote tissue regeneration and tissue repair may also depend on their paracrine effects through immune‐suppressing lymphocytes, thereby preventing over‐inflammation 27, 28, 29. To examine the effects of ASCs on the expression levels of inflammatory genes, ASC‐conditioned medium (ACM) was used to treat murine RAW 264.7 macrophage cells. The cells were pretreated with lipopolysaccharide (LPS) for 24 hrs, followed by incubation with ACM from the indicated stem cells for 24 hrs. Treatment of cells with LPS alone induced TNF‐α, IL‐6 and iNOS expressions. However, S‐ASC ACM from mice (Fig. 5A) and humans (Fig. 5B) significantly decreased the expression levels of these genes, whereas V‐ASC ACM induced expression.

## Discussion

Adipose tissue has recently drawn a great deal of attention from the stem cell research community. This may be attributed to its two major characteristics. First, adipose tissue is abundant in most normal and obese patients with OA who need stem cell therapy. In addition, thousands of people spend a significant amount of money to remove adipose tissue for medical and cosmetic causes each year, indicating that harvesting of adipose tissue for the collection of stem cells is likely accepted by the majority of the population. Second, the frequency of ASCs in adipose tissue is higher than that in bone marrow‐derived MSCs. In adipose tissue, 1–10% of nucleated cells are considered to be adipose tissue‐derived stem cells (ADSCs), whereas only 0.0001–0.01% of nucleated cells in the bone marrow are stem cells 11. Thus, a sufficient number of stem cells may be acquired from adipose tissue. All of these factors suggest that adipose tissue may become an alternative source of MSCs. Using cell lineage analysis, researchers have found a major ontogenetic difference between VS and SC WAT, and mesothelium is a source of VS adipocytes 30.

Several studies have shown that transplantation of S‐ASCs leads to regeneration of cartilage in animals and humans; however, most of these studies only focused on S‐ASCs, whereas the therapeutic capacity of ASCs from other adipose deposits, such as VS deposits, has not been addressed. Therefore, this study was designed to determine whether V‐ASCs could differentiate into mature chondrocytes and repair cartilage and improve knee function in OA. We found that intra‐articular injection of V‐ASCs did not significantly regenerate articular cartilage. However, regeneration of hyaline‐like articular cartilage after injection of S‐ASCs was clearly demonstrated in this study by IHC, histology and gene expression analysis of cartilage‐related markers. MSCs stimulate chondrocytes to proliferate and synthesize extracellular matrix, induce anti‐inflammatory cytokine production and possess immunomodulatory properties. Macrophages treated with ACM from S‐ASCs significantly decreased the levels of pro‐inflammatory‐related genes. The cells were pretreated with LPS for 24 hrs, followed by incubation with ACM from the indicated stem cells for 24 hrs. Treatment of cells with LPS alone induced TNF‐α, IL‐6 and iNOS expressions. However, S‐ASC ACM from mice and humans significantly decreased the expression levels of these genes, whereas V‐ASC ACM induced expression. Thus, S‐ASCs are more immune‐suppressive.

A 2016 report by Pak *et al*. 13 was the first study to demonstrate the possibility that ADSCs may regenerate cartilage in patients. Koh and Choi 14 used adipose SVF derived from approximately 19 g adipose tissue obtained from the knee fat pad while performing arthroscopic lavage. In addition to finding the optimal cell source for cartilage repair, it is also important to be able to predict chondrogenic outcome from a given cell population. Cell surface markers represent the easiest solution, as flow cytometry‐based assays are reproducible, automated and highly objective. Recently, it was demonstrated that SC and VS ASCs (S‐ASC and V‐ASC) express same surface markers (CD31^−,^ CD34^−^, CD45^−,^ CD73^+^, CD90^+^ and CD105^+^) and have differentiation potentials, S‐ASCs had higher capacity to proliferate and to differentiate into adipogenic lineage than V‐ASCs 31. Although the MSC markers, CD29, CD34 and Sca‐1, are available for this purpose, no well‐characterized pre‐chondrocyte markers have been identified. In this study, we found that V‐ASCs had more CD34^+^ cells than S‐ASCs, which is consistent with the high proliferative ability of V‐ASCs. However, *in vitro* chondrogenesis can be used to monitor the ability of ASCs to be effective in the treatment of OA. In our experiments, S‐ASCs differentiated into chondrocytes *in vitro*, and expressed higher levels of chondrocyte‐related genes, compared to V‐ASCs. These data provide functional and immunosuppression evidence for the usefulness of S‐ASCs in cartilage repair in OA. Therefore, *in vitro* chondrogenesis and related genes can be tested to choose the optimal cell source for treating OA.

The osteogenic potential of adipocytes derived from the VS tissue was significantly higher than those from SC adipose tissues, but the cell proliferation rate was lower than of the latter. It may be due mainly to the histological characteristics of adipose tissues and to the differences of ASC stem cell niches in different VS and SC locations. Different relationships with the superficial fascia and in fat lobule distribution among retinacula are known to affect the vascular supply and the viability and stemness of ASCs isolated from different SC depots. Different VS locations display significant biological diversities. The better the blood supply, the more the bone cells will be able to get. The number of fibroblasts will significantly affect the differentiation function of stem cells. Mixing of the stem cells derived from bone marrow and dermal fibroblasts reduced the osteogenic potential of stem cells 32. Compared with SC adipose tissue, the VS adipose tissue contains more blood supply and less fibre wrapped, which may lead to the different osteogenic potential of V‐ASCs and S‐ASCs.

In conclusion, this study showed that CD34‐positive cells with a high proliferation rate could be readily isolated from both SC and VS adipose tissue. However, the data from our *in vivo and vitro* experiments show that only SC adipose tissue appears to be a good source of MSCs for the treatment of OA. These findings may facilitate the exploration of stem cell therapy in subjects with OA. Further studies are needed to establish the criteria for protocols outlining how to induce chondrogenic differentiation *in vitro* which may predict efficacy *in vivo*.

## Funding

This study was supported by the Shanghai Zhangjiang Stem Cell Research Project (Grant No. ZJ2014‐ZD‐002).

## Conflict of interest

None declared.

## Supporting information


**Table S1.** Primers for real‐time qPCR used in this study.
