# Supplementary material for: A comparative assessment of adipose‐derived stem cells from subcutaneous and visceral fat as a potential cell source for knee osteoarthritis treatment
Source: J Cell Mol Med. 2017 Apr 4;21(9):2153–62. doi: 10.1111/jcmm.13138 (PMC5571554; doi:10.1111/jcmm.13138)
Supplement: Supplementary file 1 — Table S1. Primers for real‐time qPCR used in this study. [file JCMM-21-2153-s001.docx]

**Supplementary Table1. Primers for real-time qPCR used in this study**

| Mus/Rat  Col2a1 | GGGAATGTCCTCTGCGATGAC | GAAGGGGATCTCGGGGTTG |
| --- | --- | --- |
| Mus/Rat Sox9 | AGTACCCGCATCTGCACAAC | ACGAAGGGTCTCTTCTCGCT |
| Mus/Rat  Aggrecan | GTGGAGCCGTGTTTCCAAG | AGATGCTGTTGACTCGAACCT |
| Mus TNFα | GACGTGGAACTGGCAGAAGAG | ACCGCCTGGAGTTCTGGAA |
| Mus IL6 | CCACGGCCTTCCCTACTTC | TTGGGAGTGGTATCCTCTGTGA |
| Mus iNOS | ACATCAACCCGTCCACAGTAT | CAGAGGGGTAGGCTTGTCTC |
| Mus 18s | CGCCGCTAGAGGTGAAATTCT | CATTCTTGGCA1AATGCTTTCG |
| Human  Col2a1 | TGGACGCCATGAAGGTTTTCT | TGGGAGCCAGATTGTCATCTC |
| Human  Sox9 | AGCGAACGCACATCAAGAC | CTGTAGGCGATCTGTTGGGG |
| Human  Aggrecan | ACTCTGGGTTTTCGTGACTCT | ACACTCAGCGAGTTGTCATGG |
| Human 18s | GTAACCCGTTGAACCCCATT | CCATCCAATCGGTAGTAGCG |
